# The Impact of Long-Term Exposure to Space Environment on Adult Mammalian Organisms: A Study on Mouse Thyroid and Testis

**DOI:** 10.1371/journal.pone.0035418

**Published:** 2012-04-25

**Authors:** Maria Angela Masini, Elisabetta Albi, Cristina Barmo, Tommaso Bonfiglio, Lara Bruni, Laura Canesi, Samuela Cataldi, Francesco Curcio, Marta D'Amora, Ivana Ferri, Katsumasa Goto, Fuminori Kawano, Remo Lazzarini, Elisabetta Loreti, Naoya Nakai, Takashi Ohira, Yoshinobu Ohira, Silvio Palmero, Paola Prato, Franco Ricci, Linda Scarabelli, Tsubasa Shibaguchi, Renza Spelat, Felice Strollo, Francesco Saverio Ambesi-Impiombato

**Affiliations:** 1 DIPTERIS, University of Genoa, Genova, Italy; 2 Department of Clinical and Experimental Medicine, University of Perugia, Perugia, Italy; 3 Department of Clinical and Biological Sciences, University of Udine, Udine, Italy; 4 Institute of Pathologic Anatomy and Histology, University of Perugia, Perugia, Italy; 5 School of Health Sciences, Toyohashi SOZO University, Aichi, Japan; 6 Graduate School of Medicine, Osaka University, Osaka, Japan; 7 Graduate School of Frontier Biosciences, Osaka University, Osaka, Japan; 8 DIBIO, University of Genoa, Genova, Italy; 9 ENEA-C.R. Casaccia, Roma, Italy; 10 INRCA-IRCCS, Roma, Italy; Pennington Biomedical Research Center, United States of America

## Abstract

Hormonal changes in humans during spaceflight have been demonstrated but the underlying mechanisms are still unknown. To clarify this point thyroid and testis/epididymis, both regulated by anterior pituitary gland, have been analyzed on long-term space-exposed male C57BL/10 mice, either wild type or pleiotrophin transgenic, overexpressing osteoblast stimulating factor-1. Glands were submitted to morphological and functional analysis.

In thyroids, volumetric ratios between thyrocytes and colloid were measured. cAMP production in 10^−7^M and 10^−8^M thyrotropin-treated samples was studied. Thyrotropin receptor and caveolin-1 were quantitized by immunoblotting and localized by immunofluorescence. In space-exposed animals, both basal and thyrotropin-stimulated cAMP production were always higher. Also, the structure of thyroid follicles appeared more organized, while thyrotropin receptor and caveolin-1 were overexpressed. Unlike the control samples, in the space samples thyrotropin receptor and caveolin-1 were both observed at the intracellular junctions, suggesting their interaction in specific cell membrane microdomains.

In testes, immunofluorescent reaction for 3β- steroid dehydrogenase was performed and the relative expressions of hormone receptors and interleukin-1β were quantified by RT-PCR. Epididymal sperm number was counted. In space-exposed animals, the presence of 3β and 17β steroid dehydrogenase was reduced. Also, the expression of androgen and follicle stimulating hormone receptors increased while lutenizing hormone receptor levels were not affected. The interleukin 1 β expression was upregulated. The tubular architecture was altered and the sperm cell number was significantly reduced in spaceflight mouse epididymis (approx. −90% vs. laboratory and ground controls), indicating that the space environment may lead to degenerative changes in seminiferous tubules.

Space-induced changes of structure and function of thyroid and testis/epididymis could be responsible for variations of hormone levels in human during space missions. More research, hopefully a reflight of MDS, would be needed to establish whether the space environment acts directly on the peripheral glands or induces changes in the hypotalamus-pituitary-glandular axis.

## Introduction

Space is presently considered the “next frontier” for mankind. Besides the natural urge for exploring the unknown, a primordial characteristic of human nature, it has been envisioned that colonization of other planets may be the only chance for humankind to escape extinction, the otherwise unavoidable biological destiny for any living species.

During the last 50 years, human space exploration achieved landmark results, from the first manned orbital satellites to the Lunar landings, the construction and use of the International Space Station (ISS) and of the Hubble telescope, etc. All this has been reported in many historic newspaper headlines worldwide and in countless publications in magazines, books and scientific journals.

At variance from any other field of science however, human exposure to space environment proceeded largely by means of heroic attempts, each one of them just pushing the time limit, without any previous long-term space experimentation on animals, particularly on complex animals otherwise routinely used in “on ground” science, such as small mammals (mice, rats). Only the first pioneering and short-term space missions in the 60's involved dogs (the famous Laika) and monkeys. Those were spectacular achievements, but most scientific requirements were at that time missing (no recovery/follow-up, no statistics, no concern for animal rights, etc.). Basically, only the length of their survival was recorded, and this parameter was entirely dependent on the limits of the life-sustaining equipment and technologies, rather than to the space environment.

From then on, with limited opportunities because of the costs involved and the scarcity of space-flights compatible with scientific experiments, and despite the many unavoidable technical constrains, only *in vitro* molecular and cellular research has been and is currently performed in space. Instead, because of the many intrinsic difficulties and constraints, long-term *in vivo* studies on complex animal models have been virtually absent during the last 50 years in the international space science scenario. However, in the meantime 289 astronauts (to date) have been exposed to the extraterrestrial space environment (source: Wikipedia), several of them for many months continuously. All this without any previous *in vivo* test of the space environment as mentioned, and consequently without any previous knowledge about the long-term biological consequences and the possibly relevant yet unknown health risks for humans.

Never before the so-called “space age”, living organisms have been exposed to such alien space environment. Life itself, as we know it in our planet, evolved not taking into account the effects space environment and its variables, namely microgravity and space radiation. No countermeasures, or defense mechanisms, have been tested or refined by natural selection. For this reason, long-term animal experimentation in space, particularly involving mammals, is at this point a necessary prerequisite for the safety and health of astronauts. Many more of them will be involved in future, already envisioned longer-term human space missions, i.e. the construction and utilization of manned Lunar bases and the human exploration of distant planets (possibly asteroids, Mars and beyond).

Most of the reported pathological variations to date observed in humans, seem largely reversible upon return to Earth but 1) consequences of space exposure are still to be verified at long-term; 2) subject numbers are still too low for a correct statistical evaluation; 3) future longer-term space programs will inevitably lead to humans never returning to Earth, and eventually competing their life cycle entirely in space.

By participating to the “Tissue Sharing” team lead by R. Cancedda, we had the access to tissues and organs of mice which had been exposed to the space environment for 91 days on board of the ISS, while kept inside the “Mouse Drawer System” (MDS), a facility built by “Thales Alenia Space-Italy” for the “Agenzia Spaziale Italiana”. This is presently the longest-duration spaceflight mission ever endured by any living animal, obviously excluding human astronauts.

The authors of this paper concentrated on thyroid and testis, because of our previous long term experiences both on ground and in space research, as we had previously performed several *in vitro* studies on those tissues. It is also to be considered that the endocrine and reproductive tissues are key elements in the animal and human pathophysiology on ground and, by extrapolation, in space. Maintaining a good health in general requires the maintenance of a homeostasis which largely depends on the good and correct coordination of the endocrine system. This equilibrium is very likely to be subject to significant perturbations in space, as already proven by the several pathological variations in several body functions regularly reported by astronauts.

An additional reason stressing the relevance of space endocrine research, is its profound influence on mental conditions, and ultimately on mood, personality and performance of human subjects. This may become particularly relevant during long-term space missions because of several considerations, too many to be listed here. As an example, suffice to just mention here a few key words: confined habitat; teamwork; human/computer/machine interaction; attention span; goal focusing; coordination; sleep pattern; depression, etc.

Thyroid has proven to be particularly sensitive to the long-term effects of radiation exposure, as proven in the countless follow-up studies of human subjects exposed to sublethal radiation doses (Hiroshima, Nagasaki, Marshall Islands, Chernobyl, etc.). Because of its profound effect on metabolism and on several relevant body functions (heart, brain, etc.), following our several *in vitro* space research experiences, we felt it would be important to use this rather unique opportunity of *in vivo* space research, in which thyroid glands from wild type (WT) and transgenic (TG) mice (the latter over-expressing pleiotrophin (PTN), one of the osteoblast stimulating factor-1) could be examined, following a remarkably long-term exposure to space environment.

Testis has also been chosen in the present study, because its relevance as endocrine organ, and also because of its key role in the reproductive system. Spaceflight has also been shown to significantly affect the physiology of the testis. The dual functions of the adult testis, namely, the production of spermatozoa and the secretion of testosterone, are both dependent on stimulation by the pituitary gonadotropins, Follicle-Stimulating Hormone (FSH) and Luteinizing Hormone (LH), which are secreted in response to hypothalamic Gonadotropin-Releasing Hormones (GnRH). Testosterone, which is essential for promoting spermatogenesis, is secreted by the adult Leydig cell under LH stimulation, and acts via androgen receptors mainly located in Sertoli cells. FSH instead acts through specific receptors located on the plasma membrane of Sertoli cells (see review [1]).

In addition to the roles of gonadotropins and androgens in the initiation and maintenance of spermatogenesis, the function of autocrine and paracrine regulatory factors must also be considered. Among these, cytokines play a relevant role in the development and normal function of the testis, as well in inflammation (see review [2]).

In the present study, the influence of weightlessness on the endocrine regulation of the male gonad function was investigated. To this aim, the mRNA expression of receptors involved in the response to GnRH and androgens, namely the FSH Receptor (FSHR), LH Receptor (LHR) and Androgen Receptor (AR), was evaluated, by quantitative RT-PCR, in the testis of mice under different gravity conditions (0 g and 1 g). In addition, as a marker of inflammation, the expression of mRNA for interleukin-1beta (IL-1beta) was evaluated.

To date, the information on the effects of spaceflight on mammalian reproductive function, such as sperm number, is limited. Fedorova [3] reported that 22 days of spaceflights on Cosmos 110 caused an increased sperm abnormalities in dogs. Rats flown on Spacelab 3 for 7 days and Cosmos 1887 for 13 days showed a reduction in spermatogonia [4], [5]. Although spermatogenesis was not affected in rats exposed to space environment for less than 14 days [6], [7], plasma and testicular testosterone contents were markedly decreased [6]. Amann et al [6] speculated that long-duration spaceflight might result in impairment of spermatogenesis, because the testosterone level in the flight rats was close to the minimal amount obligatory for normal spermatogenesis [8].

In the ground-based studies, hindlimb suspension and radiation have been widely used as the models for exposure to microgravity and space radiation, respectively. Short-term (approximately 1–2 weeks) hindlimb suspension resulted in a reduction in testosterone concentration [6]–[11], sperm motility [9], and spermatogenesis [6] in rodents. Decrease in spermatogenesis was also observed in rats after long-term (6 weeks) hindlimb suspension [12]. In addition, mice exposed to cosmic type radiation showed a significant decrease in spermatogonia [13], [14].

However, there are no studies investigated the effects of long-term spaceflight on the mammalian reproductive function. Moreover, effects of over-expression of PTN, one of the osteoblast stimulating factor-1, on reproductive function are still unclear. Therefore, the present study was performed to investigate the effects of long-term spaceflight and over-expression of PTN on mouse reproductive function.

Out of the 6 animals sent to Space inside MDS on board of the Shuttle Discovery and then transferred to the ISS, only 3 returned to Earth alive after their 91 days space mission. Two of them were TG, and one WT. Normally this low number of experimental animals would be considered inadequate for conducting scientific analyses. But because of the exceptionality of the experimental substrate (animals kept in space for the longest- time ever, as already mentioned) and because of the impossibility to repeat such an experiment in a reasonable time (a reflight of the MDS, although strongly requested by the “tissue sharing” team and by all the space scientific community at large, is still to be approved by the international space Agencies), we decided to go on with the experimentation and in writing this report. We believe that the data here reported, will already answer to some of the questions regarding the consequences of spaceflight in animal endocrine physiopathology, and will also contribute to the possibility of repeating and expanding this kind of space research in the near future.

## Results

### A. Thyroid

#### 1. Changes of thyroid tissue morphology in microgravity

**Figure 1:**
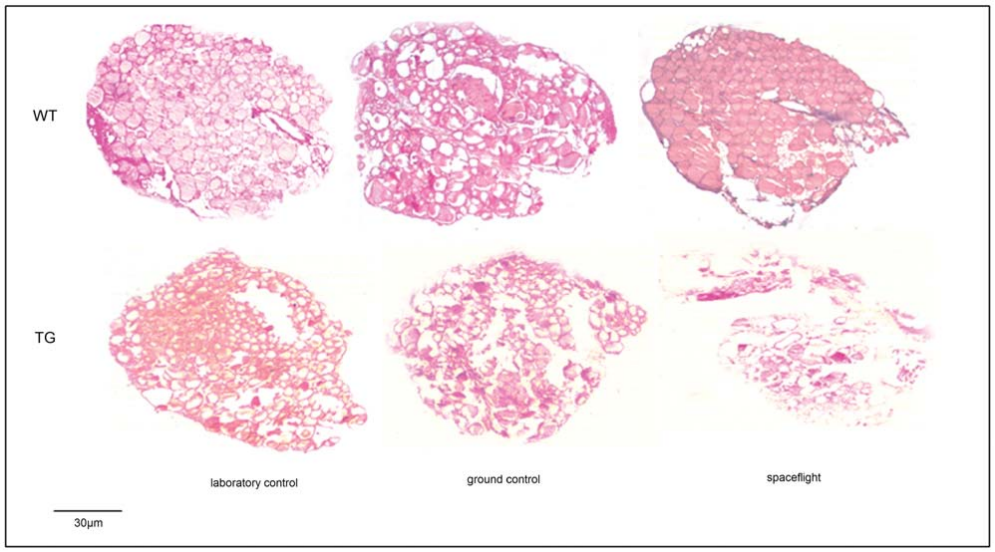
Morphology analysis of thyroid tissues of WT and TG mice maintained in laboratory, in vivarium cages (laboratory control) and in MDS (ground control), from either the on-ground or the spaceflight group. Hematoxylin-eosin staining, 4× Magnification.

In order to study the effect of exposure to microgravity on thyroid gland, we have first investigated the possible changes of thyroid tissue structure. Microscopy analysis, performed on histological microsections of WT laboratory control subjected to hematoxylin-eosin staining, showed that the surface area was 13.7±2.1 mm^2^. As is usually known [15], the size of the follicles was not at all homogeneous, larger follicles were found in the periphery and smaller ones were found centrally (Fig. 1). In all analyzed samples, no significant changes were noted in the major and minor axis of the histological section. Thus the surface areas were substantially similar but some structural differences were evident. In the thyroid gland of WT ground control, follicles showed variable size and spatial orientation. In contrast, spaceflight animals had a more homogenous thyroid tissue structure, with ordered follicles and reduction of interfollicular space. The thyroid glands from TG mice displayed the following atypical histological features: 1) a marked decrease of follicular surface; 2) abnormally large clear areas, progressively larger in the order of: laboratory control>ground control>spaceflight animals (Fig. 1).

**Figure 2:**
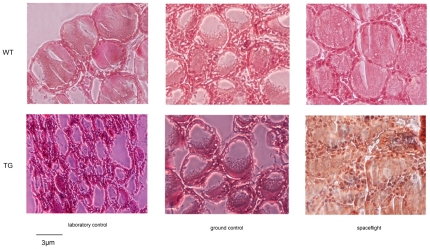
Morphology analysis of thyroid tissues of WT and TG mice maintained in laboratory, in vivarium cages (laboratory control) and in MDS (ground control), from either the on-ground or the spaceflight group. Hematoxylin-eosin staining, 40× Magnification.

Figure 2 shows section details to highlight the follicular pattern, i.e. structures contained colloid and surrounded by a single layer of thyroid epithelial cells or thyrocytes. In WT animals, the basal pole of thyrocytes delineate a continuous rim that becomes very clear in follicles of spaceflight animals. In these animals, follicular thyrocytes were thicker and the nuclear volumes appeared to increase; consequently the thyroid epithelium vs. colloid volumetric ratio changed. The follicles of TG laboratory and ground animals were characterized by poorly developed follicles that were heterogeneous because of the variable size of both cells and colloidal spaces. In the spaceflight animals, both WT and TG, the size of follicles was greatly heterogeneous (Fig. 2).

#### 2. The microgravity influences thyroid gland function

We have tested the possibility that the structural modifications of thyroid gland in real microgravity environment could be related to its functional changes. Table 1 shows the basal and thyrotropin (TSH)-stimulated cAMP production in thyroid gland. In all analyzed samples, the effect of 10^−7^M TSH treatment was higher than that obtained with 10^−8^M TSH. The maximum level of both basal and TSH-stimulated cAMP production in WT and TG animals was observed in the space-exposed animals. The effect was more marked in WT animals where the level of cAMP produced with 10^−7^M TSH in spaceflight animals was 5.78 and 4.44 folds higher than that obtained in laboratory control and ground control, respectively.

**Table 1:** Effects of long-term exposure to real microgravity environment on cAMP production after thyrotropin (TSH) treatment of thyroid tissue in wild type and transgenic mice.

	laboratory control			ground control			spaceflight		
WT	C	10^−8^	10^−7^	C	10^−8^	10^−7^	C	10^−8^	10^−7^
1	1.14	10.11	33.12	–	–	–	9.54	20.76	248.48
2	2.61	18.24	26.22	3.70	13.22	56.00	–	–	–
3	4.98	7.36	72.16	–	–	–	–	–	–
mean ± SEM	2.91±0.87	11.90±2.67	43.83±11.60	3.7	13.22	56.00	9.54	20.76	248.48

TG	C	10^−8^	10^−7^	C	10^−8^	10^−7^	C	10^−8^	10^−7^
1	3.91	16.33	60.51	1.40	19.58	76.75	5.93	48.02	136.04
2	1.87	9.66	52.67	5.34	14.63	50.40	1.32	57.05	125.82
3	3.22	17.13	67.33	–	–	–	–	–	–
mean ± SEM	3.00±0.49	14.37±1.95	60.50±3.48	3.37±1.40	17.10±1.75	63.57±9.34	3.62±1.63	52.53±3.20	130.93±3.62

WT, wild type; TG, transgenic. The data are expressed as pmol cAMP/mg protein. C, control sample without thyrotropin treatment; 10^−8^ and 10^−7^, thyrotropin concentrations.

**Figure 3:**
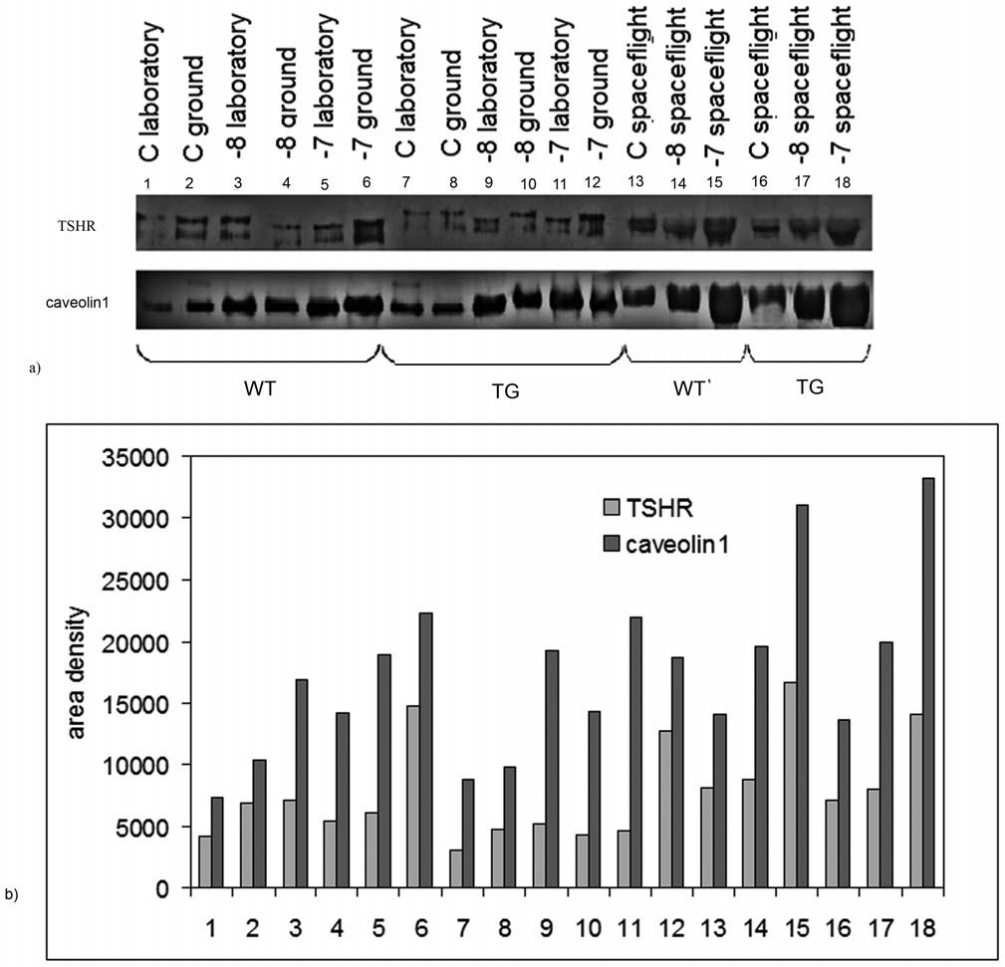
Thyrotropin receptor (TSHR) and caveolin1 in thyroid tissues of WT and TG mice maintained in laboratory, in vivarium cages (laboratory control) and in MDS (ground control), from either the on-ground or the spaceflight group. The study was performed by immunoblotting analysis by using specific antibodies. The position of the 50 kDa protein for TSHR and 22 kDa for caveolin1 was indicated in relation to the position of molecular size standards. The area density was evaluated by densitometry scanning and analysed with Scion Image programme.

Thus, we next asked whether increasing response to TSH treatment in space-flown animals could be due to the modification of TSH receptor (TSHR). To address this question, the immunoblotting analysis was performed using specific antibodies against TSHR and caveolin1. Our results demonstrated that the long-term exposure to real microgravity environment induced an increase of TSHR band, corresponding to 50 kDa apparent molecular weight, in the TSH-untreated samples (C) with respect to both laboratory and ground controls either in WT or in TG animals (Fig. 3a). The band area density analysis demonstrated that the increase of TSHR in space environment was equal to 95% and 18% in WT animals whereas 129% and 70% in TG animals vs. the laboratory and ground controls, respectively (Fig. 3b).

Similarly the band of caveolin1, corresponding to 22 kDa apparent molecular weight, showed a higher immunopositivity in the thyroid of spaceflight animals with respect to laboratory and ground controls than the same proteins present either in WT or in TG animals (Fig. 3a). The band area density analysis demonstrated that the increase of caveolin1 in space environment was equal to 92% and 37% in WT animals whereas 55% and 38% in TG animals in comparison with laboratory and ground controls, respectively (Fig. 3b).

The treatment of the samples with 10^−7^M TSH increased the TSHR and caveolin1 bands in all three experimental model: laboratory control, ground control and spaceflight animals (Fig. 3a,b).

**Figure 4:**
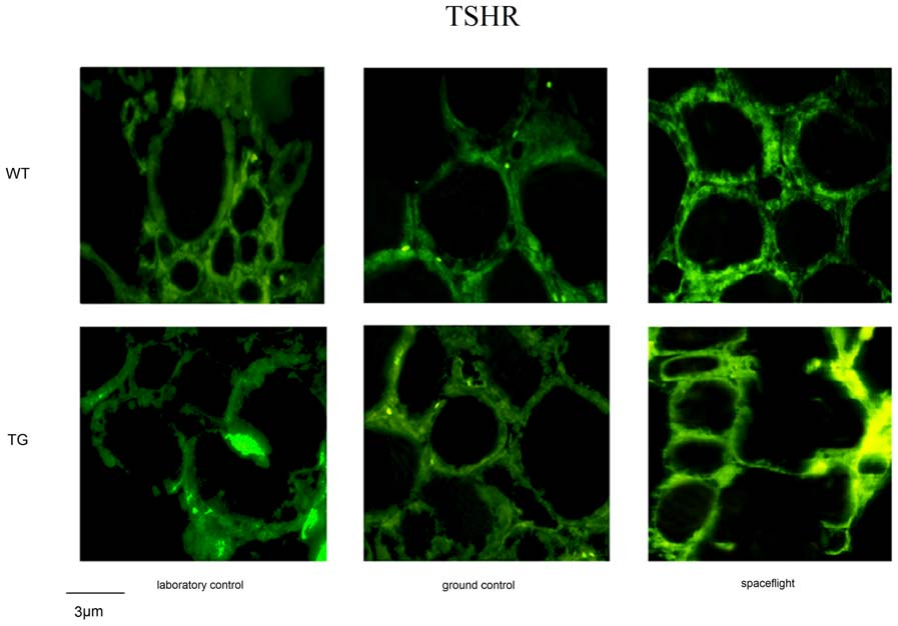
Fluorescence immunostaining of thyrotropin receptor (TSHR), using anti-TSHR antibody in thyroid tissues of WT and TG mice maintained in laboratory, in vivarium cages (laboratory control) and in MDS (ground control), from either the on-ground or the spaceflight group.

**Figure 5:**
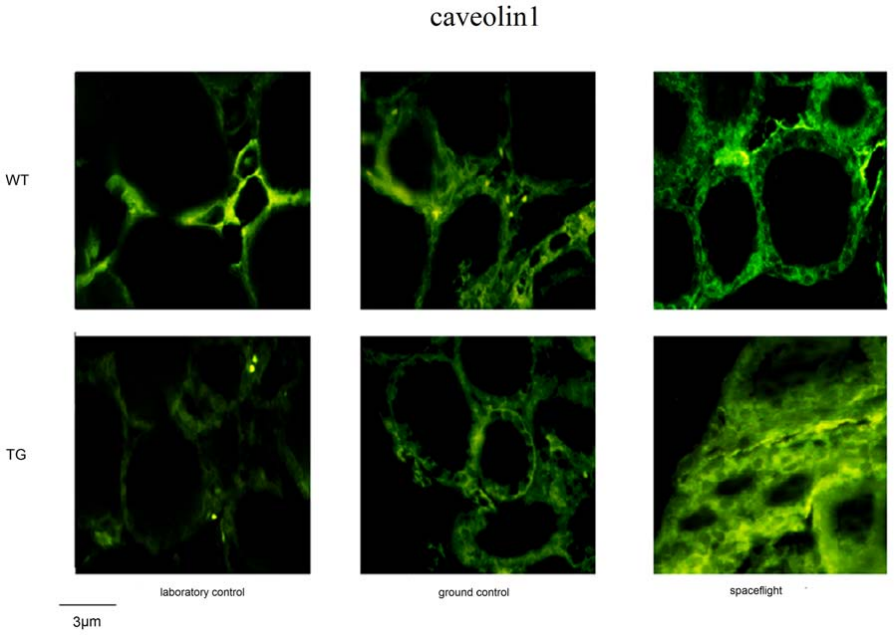
Fluorescence immunostaining of caveolin 1, using anti-caveolin 1 antibody, in thyroid tissues of WT and TG mice maintained in laboratory, in vivarium cages (laboratory control) and in MDS (ground control), from either the on-ground or the spaceflight group.

Immunofluorescence analysis confirmed the increased expression and highlighted a different distribution of either TSHR or caveolin1 in the spaceflight animals. In WT animals the TSHR present in the thyroid gland of laboratory control was distributed uniformly in the cellular component of thyroid follicles (Fig. 4). In ground control, receptor fluorescence was particularly intense at the basal pole of thyrocytes and clearly highlights the follicular borders. In spaceflight sample, intense protein localization was observed at the cell-cell junctions, in the cell membrane but no accumulation was found in the nuclei (Fig. 4). Caveolin 1 had an irregular distribution in the two controls and was highly consistent with the localization of the TSHR in the spaceflight sample (Fig. 5). The thyroid glands of the three experimental models from TG mice displayed very different distribution of TSHR with respect to WT mice (Fig. 4). Differently, caveolin1 had many similarities with regard to their respective WT samples, the differences being due to the different shape of the follicles rather than to the protein distribution (Fig. 5).

### B. Testis

#### 1. Histomorphology

**Figure 6:**
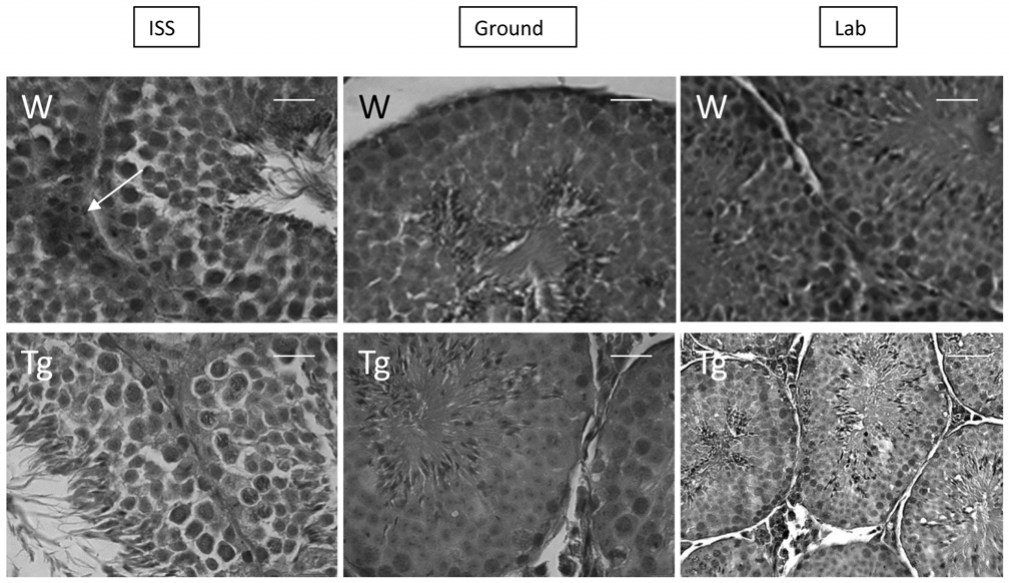
Hematoxylin-eosin staining of mouse testis slices. a) ISS wild, d) ISS transgenic; b) Ground control wild, e) Ground control transgenic; c) Lab control wild, f) Lab control transgenic. The controls show a regular tubular architecture. In a) and d) some tubular lumina contain sloughed cells. The interstitial tissue is abundant. displaying inflammatory exudates (arrows). Scale bar = 30 (a, b, c, d, e) and 65 µm (f).

In the present study, the testicular sections of both ground and laboratory controls (both WT and TG mice) were very similar. Histological examination of the testes of the control groups revealed regularly configured seminiferous tubules. The tubules were rounded or oval in cross-sections, apparently of the same diameter. The tubules were lined with a complex stratified epithelium composed of two cell types: spermatogenic and Sertoli cells. The spermatogenic cell-populations were intact and of average thickness, and appeared in various stages of maturation (Fig. 6b, c, e, and f). They were typically arranged from the basal lamina to the tubular lumen in succession as spermatogonia, spermatocytes, spermatids and spermatozoa. The spermatogonia were small cells located in the basal compartment immediately above the basal lamina. The remaining spermatogenic cells were located in the adluminal compartment. The primary spermatocytes were identified as the largest cells with large nuclei and rounded outlines. The secondary spermatocytes were not frequently seen. The spermatids were observed as small cells close to the tubular lamina that contained the tails of the spermatozoa. The Sertoli cells were detected at intervals between the spermatogenic cells. The Leydig cells were found in the interstitial stroma between and around the seminiferous tubules. They were found individually and in small groups closely related to blood capillaries.

The testes of the spaceflight animals showed degenerative changes. These included disorganization and a slight reduction in the thickness of the spermatogenic cells (Fig. 6a, d). In addition, sloughing of cells in the lumen, separation of germ cells from the basal laminae and vacuolation of the germinal epithelium were observed. The interstitial tissue displayed inflammatory exudates. More seminiferous tubules were shrunken, obviously distorted and showing severe diminution of the spermatogenic cell-masses. The testicular degeneration was very severe, to the extent that some tubules appeared almost devoid of spermatogenic cells except for a few spermatogonia, but the tubular degeneration was not homogenous in distribution as some tubules were more adversely affected than other tubules. In some tubules, the germ cells showed shrinkage of their cytoplasm resulting in enlargement of the intercellular spaces. No significant differences were observed between WT and TG mice.

### 2. Immunohistochemistry

**Figure 7:**
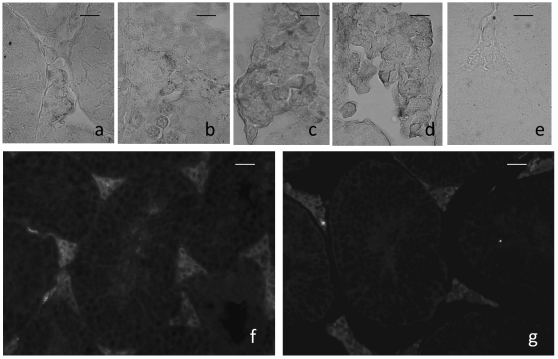
Immunofluorescent reaction for 3β- steroid dehydrogenase (a, b, c, d, e) in the intertubular tissue of wild type and transgenic mice housed in International Space Station (ISS) (a,b respectively) and in ground control mice (c,d respectively). No immunoreactions were present in control section (e) in which one step of the reaction was abolished. Scale bar = 25 µm. Immunohistochemical reaction for HSP70 in testis of WT (f) and TG mice (g) kept on the ISS are also shown. A slightly more intense stain was visible in f). Scale bar = 50 µm.

Immunohistochemistry for 3β-steroid dehydrgenase (3β-HSD) and 17β-steroid dehydrogenase (17β-HSD) showed similar localization in Leydig cells in all the animals studied. Therefore, immunoreactions of the Leydig cells in spaceflight WT and TG animals were less numerous in the ISS animals if compared with ground and laboratory control mice (Fig. 7c, d).

Heat Shock Protein 70 (HSP70) immunohistofluorescence was not detectable in ground and laboratory controls, while a light reaction was noted in the TG mice (Fig. 7g) and a more bright immunoreactions was shown in testis from WT mice (Fig. 7f). The immunopositivity was localized in the interstitial tissue.

### RT-Q-PCR

**Figure 8:**
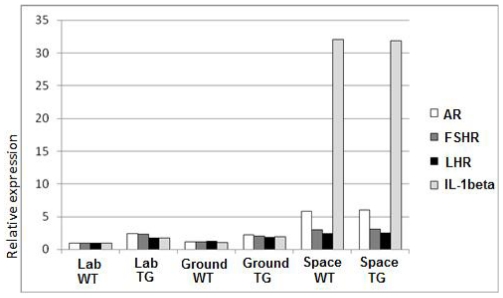
Mean relative expressions of androgen receptor (AR), follicle stimulating hormone receptor (FSHR), lutenizing hormone receptor (LHR), and interleukin-1β (IL-1β) in testicular tissue. They were quantified by real-time RT-PCR. Lab, laboratory vivarium control; ground, housed in mouse drawer system on the ground; space, spaceflight; W = WT and T = TG mice, respectively.

The effects of exposure to microgravity on the expression of AR, FSHR, LHR and IL-1 beta were evaluated and the results are shown in Figure 8. Transcription of these genes was comparable in ground and laboratory controls, with slightly higher levels of mRNA transcripts in TG with respect to WT mice. Exposure to microgravity did not affect the expression of LHR in both WT and TG mice, whereas it induced an increase in FSHR expression in WT. On the other hand, higher levels of mRNA transcripts for AR were observed in testes of spaceflight WT and TG mice. Moreover, a particularly higher expression of IL-1β was observed in space-flown samples.

### 3. Sperm counts

**Figure 9:**
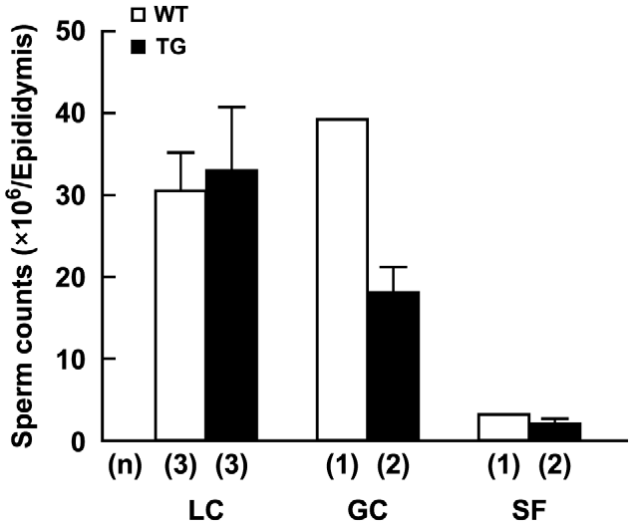
Effects of 3-month exposure to space environment on the epididymal sperm number in wild type (WT) and transgenic (TG) mice. Mean±SEM. n = 1 in GC and SF, and 3 in LC of WT, and n = 2 in GC and SF, and 3 in LC of TG mice, respectively. LC: laboratory vivarium control, GC: ground control housed in mouse drawer system on the ground, and SF: spaceflight group.

Figure 9 shows the number of epididymal sperm across the experimental groups. Statistical significances were not examined, because the number of experimental animals was limited. The Mean Sperm Number (MSN) in the laboratory controls was identical among WT (30.3±5.0×10^6^) and TG (32.7±7.9×10^6^) mice. The MSN in the ground control group of WT mice (39.3±10×10^6^) tended to be greater than the one in the laboratory control group (+23%). However, the MSN in the ground control group of TG mice (17.9±3.0×10^6^) tended to be less than the one in the laboratory control (−45%). Very low sperm numbers were noted in both WT and TG spaceflight mice (−90%, −94%, −92% and −89% vs. laboratory and ground controls, respectively).

## Discussion

We are well aware that the number of animals tested in this study, one Wild Type and two Transgenic, is a limiting factor for reaching statistically significant experimental results. However:

1. the choice of including Transgenic animals in the MDS experiment is not ours, but due to the choice of the majority of investigators within the “Tissue Sharing” community, as from the companion article “The Mice Drawer System (MDS) Experiment and the Space Endurance Record-Breaking Mice” by Cancedda et al.” to be published in the same special issue of PLoS ONE;
2. for all we know, the differences between the Wild Type and the Transgenic animals, due to a bone metabolism - related transgenic gene, should not concern the thyroid or the testicular tissues, nor the thyroid- or testis-specific functions. Therefore, based on all the present knowledge the three surviving animals could be considered equivalent for our purposes. Based on this assumption, we decided to proceed with the tissue evaluation of all survivers, as from our original protocol;
3. This specific aspect of our study, i.e. the limited number of animals on one side, and the unique opportunity to study animals exposed for a record-breaking time length to the Space environment on the other side, has been addressed to in the above mentioned article from Cancedda et al., in this same issue of PLoS ONE. There, the Authors state (quote): “Undoubtedly, the limited number of mice (three wild type and three PTN-tg mice) that the MDS payload could house during the flight, together with the fact that unfortunately only three mice (one wild type and two PTN-Tg mice) survived to the 91-day spaceflight, represents a critical aspect of the experiment, especially for the statistical reliability of the collected data. However the MDS experiment was a unique opportunity to study the microgravity long-term exposure effects on several tissues of an animal model and to collect interesting observations that could prepare the field to future experiments.”

All this considered, we report here for the first time the effect of long-term exposure to real microgravity environment on thyroid function *in vivo*. The initial step of the thyroid gland stimulation is the TSH-TSHR interaction on the basolateral membrane of thyrocytes. Upon binding of TSH, cAMP produced at the basolateral membrane diffuses through the cytosol to activate cytosolic protein kinase A (PKA) I and PKA II, mainly located in the Golgi complex; PKAs in turn phosphorylate targets localized in different cellular compartments [16], [17]. We have recently observed that thyroid cells in culture delay cell growth during the space missions [18] with the decrease of cAMP level for the insensitivity of TSHR to TSH [19].

The experimental design used for the study is complex, because: 1) *in vivo* the thyroid function is regulated by hypothalamic-pituitary-thyroid axis; 2) the animals began the experimental protocol at an early age and ended in adulthood and thyroid function is related to age [20]. At the re-entry of space mission thyroid lobes were excised and analyzed. Treatment *in vitro* with TSH induced the release of cAMP from thyroid lobes, resulting in the increase of cAMP concentration in the incubating medium. Our results indicated clearly that 10^−7^ M TSH stimulated cAMP release in the spaceflight animals with a value higher of that obtained in the control animals. The structure of thyroid follicles appeared more organized and TSHR was more expressed. The size and shape is of fundamental importance, as modifications of these parameters are expected to alter the gradients of cAMP and the properties of signaling cascades, including cAMP and other soluble messengers [21], [22]. TSHR is a G protein-coupled receptor [23] associated with no-raft and raft fractions of cell membrane rich in caveolin1 content [24]. TSHR-rafts complexes are regulated by TSH [25]. After TSH exposure with consequent signal transduction initiation and cell activation, the raft-TSHR complexes disappear from the cells surface, probably because TSH stimulates the formation of monomers allowing their rapid exit [25].

TSHR continues to signal to adenylyl cyclase after internalization into a pre-Golgi compartment, and the location of TSHR-cAMP signaling affects the spatial patterns of the downstream signals [26]. Our data highlighted a similar localization of TSHR and caveolin1 in thyroid follicular cells of spaceflight animals, in different way with respect to control samples. On the basis of all findings obtained in the present study, we suggest that long-term exposure to real microgravity environment induces structural/functional changes of thyroid gland.

It can be hypothesized two different possibilities that could justify this observation: 1) because of the duration of the experiment, the animals were analyzed when they became adults with thyroid activity lower than that of the juvenile period [27]. It possible that space environment delays the aging and maintain thyroid activity higher than that of controls; 2) thyroid changes are expression of the response to stress. The stress conditions are associated with decreases in peripheral T3, T4 and TSH when compared with controls [28]. It is possible that the increase of TSHR in thyroid of spaceflight animals is a compensatory mechanism to stress that allows the strong response to TSH after stimulation of lobes *in vitro*. This last hypothesis could explain why the results of the ground control animals are intermediate between those of laboratory control animals and those of spaceflight animals. Perhaps the confined environment of the ground control samples could represents a stress factor and then the data in spaceflight animals are due to the sum of stress of confined environment and space environment.

The data obtained in TG animals could be due to fact that osteoblast stimulating factor is an extracellular matrix-associated protein [29] that may be present also in the thyroid matrix. Thus, the protein overexpression could induce disorganization of the structure. It is evident from our data that the effects of space environment appear attenuated in TG animals with respect to WT animals.

As mankind enters the space age, it may become a critical problem whether humans will be able to maintain a normal life cycle, including reproduction, in the extraterrestrial environment. Reproduction experiments in real microgravity environments have been carried out with invertebrate and vertebrate specimens. A complete life cycle has been observed in *Drosophila melanogaster* [30]. Normal pregnancy and normal early embryogenesis occurred in killifish [31]. In mammals however, reports regarding the effect of spaceflight on spermatogenic ability are still scarce and insufficient. In microgravity environment, we observe a significant body fluid shift, as blood distributed in the upper half of the human body increases by about 2 liters, causing a facial edema and cardiovascular responses, as well as disturbance in hormones and reproductive organs [32].

The decrease in testosterone level observed in rodents after 14 days of tail suspension may be correlated to the reduced testicular blood flow in association with the cranial shift of body fluid. Tash et al. [33] demonstrated that after an early decrease in testosterone level, there is a recovery by the end of 6 weeks of tail suspension. A compensatory mechanism may thus be hypothesized in long-term experiments.

An impairment in spermatogenic function in tail suspended mice was observed. Similar results were obtained after testosterone affecting drug exposure [34] or after trauma [35]. After 6 weeks of hindlimb suspension with inguinal canal ligation, rats showed a significant decline in testicular weight compared with the control groups. Histologically, few abnormalities were observed in some seminiferous tubules in 1-week hindlimb-suspended group. Spermatogenesis was significantly reduced after a 6-week hindlimb-suspension marked by atrophy of the testes and loss of all germ cells, except a few spermatogonia [36].

In the present work, exposure to microgravity has led to degenerative changes in the seminiferous tubules as testified by the altered tubular architecture and reduction in the number of spermatogenic cells. A reduction in blood supply of the testes may have a role in blocking spermatogenesis. A decreased blood flow rate of 30% causes a significant increase in the number of spermatogenic cells showing apoptosis [37].

Synthesis of testosterone is the result of an enzymatic cascade of steroid dehydrogenases. The key enzymes are 3β-steroid dehydrogenase (3β-HSD) and 17β-steroid dehydrogenase (17β-HSD). 3β-HSD/Δ^5^- Δ^4^ isomerase catalyses the formation of Δ^4^-3 ketosteroids from Δ^5^-3β-hydroxysteroids (leading to the formation of progesterone and androstenedione). The enzyme is an obligated step in the biosynthesis not only of androgens and estrogens, but also of mineralcorticoids and glucocorticoids. A 17β-HSD dehydrogenase catalyses the formation of testosterone from androstenedione. The low presence of the enzymes 3β-HSD and 17β-HSD observed after exposure to real microgravity was correlated with the histological abnormalities observed in testes from ISS animals.

Heat shock proteins (HSPs) are a family of chaperon proteins that are induced in cells in response to environmental, physical and chemical stresses. They limit the consequences of damage and facilitate cellular recovery and survival. HSPs are a large family named accordingly to their size and include HSP100, 90, 70, 60 and 27. The function of HSPs encompasses an anti-apoptotic role. Hsp70 proteins can act to protect cells from thermal or oxidative stress. These stresses normally act to damage proteins, causing partial unfolding and possible aggregation. By temporarily binding to hydrophobic residues exposed by stress, Hsp70 prevents these partially-denatured proteins from aggregating, and allows them to refold [38]. Our data suggest that HSP70 is present only in testes interstitial tissue mainly in spaceflight WT. A similar pattern was observed in rat testes after formaldehyde inhalation [39] and in testicular slices subjected to simulated microgravity [40].

The AR, also known as NR3C4 (nuclear receptor subfamily 3, group C, member 4), is a type of nuclear receptor that is activated by binding to either the androgenic hormones testosterone or dihydrotestosterone in the cytoplasm and is then translocated into the nucleus [41]. In human spermatogenesis arrest syndrome a high androgen receptor immunoexpression, when compared with normal testis, has been demonstrated [42]. Our data, showing that microgravity induces morphological alterations of the testis and impaired testosterone metabolism, as well as increased transcription of AR, indicate impairment of spermatogenesis.

FSH has multiple and changing roles in the regulation of spermatogenesis. The first function of FSH is to increase the number of Sertoli cells by stimulation of their mitotic activity. FSH-receptor is a transmembrane receptor that interacts with FSH and represents a G Protein-Coupled Receptor (GPCR). Its activation is necessary for the hormonal functioning of FSH. FSHR activity enhances Sertoli and spermatogenic development in normal testes, but has limited ability to maintain spermatogenesis during gonadotropin deficiency [43]. The small increase in transcription of FSHR observed in WT mouse testes exposed to real microgravity, seems to support this hypothesis.

The LHR is a transmembrane receptor found in the ovary, testis and extragonadal organs like the uterus. The receptor interacts with both LH and hCG and represents a GPCR. In the male the LHCGR has been identified on the Leydig cells that are critical for testosterone production, and support spermatogenesis [44]. Our data indicate that transcription of LHR was unaffected by exposure to microgravity.

IL-1β is a member of the cytokine family and an important mediator of the inflammatory response. IL-1b does not appear to be produced in significant amounts in the normal testis. However, a different situation occurs in infection and inflammation where an upregulation of inflammatory IL-1b might contribute to the injury of testicular tissue [45].

We observed high levels of IL-1β mRNA transcripts in testes from ISS animals. These data well correlate with the histological observation where a large inflammatory region was present in the intertubular zone.

Taken together, our results suggest that spermatogenesis is affected by a prolonged exposure to real microgravity, and that decrease in blood volume and/or changes in hormonal secretion are probably responsible for the reproductive function impairment.

This is the first study reporting the effects of long-term spaceflight and over-expression of PTN on mouse epididymal sperm number. The data obtained in the present study indicated that living in space on the ISS for 3 months resulted in a marked reduction of the number of epididymal sperm in mice regardless of their phenotypes. The results obtained in the present study confirmed the previous reports that 7–13 days of spaceflight caused a reduction in spermatogonia in rat testes [4], [5] and that an absence of mature sperm in epididymides was observed in rats after long-term (6-week) hindlimb suspension [12].

Effects of spaceflight on the ability for reproduction in mammals, including humans, are still unclear. During the stay in space, astronauts and experimental animals are exposed to radiation and microgravity. It is well-known that exposure to radiation results in damage to the genes in various cells. It was reported that mice exposed to radiation in ground-based study showed a loss of spermatogonia, suggesting that exposure to radiation in space may cause the dysfunction of reproductive ability [13], [14]. Tash et al [12] speculated that the absence of gravity may cause the relocation of testes into the abdominal space, resulting in an elevation of testicular temperature. Long-term testicular hyperthermia caused by the relocation into the abdominal space could influence a reduction in sperm number [46]. Such phenomena may induce an impairment of reproductive organs [10], [12]. Therefore, it is suggested that such factors may cause the reduction of epididymal sperm by long-term spaceflight, although it is still unclear which of the radiation or microgravity more strongly influences the reproductive system.

There were no differences in the mean sperm number between WT and TG mice in the laboratory control group. The ranges of epididymal sperm number in WT and TG mice were 22.1 to 39.3×10^6^ and 21.2 to 47.8×10^6^, respectively. However, 3-month housing in MDS in 1-G environment tended to induce different response in WT and TG mice. The number of sperm in one WT ground control mouse (39.3×10^6^) was at the upper end of that in the WT laboratory control mice. The sperm number of the two TG ground controls (21.0 and 14.9×10^6^) tended to be less than the range of the TG laboratory controls. Therefore, these results indicate that lower number of sperm seen in the spaceflight group is not caused by housing in MDS.

In conclusion, our results indicate that long-term exposure to microgravity environment may induce the reproductive dysfunction in male mammals. Our data also suggested that over-expression of PTN might not affect the ability of male reproductive function. However, further study will be needed to clarify the effects of spaceflight on the male reproductive ability, because only 3 mice returned alive to Earth in the present flight mission.

In overall, our study of thyroid and testis functions in mice exposed to space environment over a period of more than 90 days, indicated that several changes occur in relevant endocrine organs under the control of the pituitary gland. Those changes significantly affect the endocrine homeostasis of the body, as well the reproductive function.

We are aware that such changes need to be further confirmed, and clarified from a cellular and molecular point of view. Furthermore, the key element (microgravity, radiation?) responsible for these relevant modifications need to be clearly identified. We do hope that more opportunities for this kind of *in vivo* space experimentation, ideally a reflight of the MDS, should be made available for our scientific community in the near future.

These kinds of observation may be instrumental for developing protective measures and countermeasures, to be adopted for the health and safety of human astronauts, prior to expose them to unsustainable risks during long-term space flight missions. As a fallout from space research, such measures and countermeasures could be equally relevant for the health and safety of all individuals exposed on Earth to extreme living and/or working conditions.

## Materials and Methods

Experimental design and animal care. In all phases of the experiment (pre-flight, during the flight and post-flight) handling of animals was in accordance with the principles expressed in the “Guide for the care and the use of laboratory animals” (Office of Science and Health Reports of the USA National Institute of Health, Bethesda, USA). The approval of the MDS experiment was requested and obtained by the American Institutional Animal Care and Use Committee (IACUC protocol n° FLT-09-070 - KSC) as well as by the Ethics Committee of the Animal Facility of the National Institute for Cancer Research (Genoa, Italy) and by the Public Veterinary Health Department of the Italian Ministry of Health (Ministero del Lavoro, della Salute e delle Politiche Sociali prot n° 4347-09/03/2009-DGSA.P.).

The authors of this article were not directly involved in/responsible for designing and/or executing the animal maintenance part of the experiment. Instead they were allowed access to the mice at the end of the flight mission and of the ground control experiments and participated in the specific tissue collection. Additional information about the MDS hardware adopted for housing the animal in space and the animal behavior during the flight, are reported in the companion article “The Mice Drawer System (MDS) Experiment and the Space Endurance Record-Breaking Mice” by Cancedda et al.”

The spaceflight experiments were carried out using male C57BL/10J mice (8 weeks old at launch). WT and PTN TG mice (n = 3 each) were individually housed in the Mouse Drawer System (MDS), a 11.6×9.8×8.4 cm payload developed by Thales-Alenia Space Italy [47]. PTN transgenic mice were utilized to investigate the possibility of this osteogenic factor for protection of osteoporosis [48]. Food and water were supplied *ad libitum*. The MDS, loaded with 3 WT and 3 TG mice, was launched in the Space Shuttle Discovery within the Space Transport System (STS)-128 mission, on August 28, 2009. It was then housed in the Japanese Experimental Module (Kibou) on the ISS until its return to the Earth by Space Shuttle Atlantis (STS-129 mission) on November 27, 2009. Only 1 wild type and 2 transgenic mice returned to the Earth alive after 91 days of space flight. Thyroids and testis, including epididymis, and were sampled bilaterally from each mouse killed by inhalation of carbon dioxide at the Life Sciences Support Facility of Kennedy Space Center within 3–4 hours after landing and either processed or frozen immediately, according to the various experimental protocols. The procedure was approved by the IACUC protocol n° FLT-09-070(KSC).

After the spaceflight experiment, the on-ground control experiment was carried out at the Vivarium of the Advanced Biotechnology Center in Genoa, Italy. One group of mice of the same species, sex, and age were housed in MDS for 3 months as the ground controls (GC). Another group of mice were housed in normal vivarium cage as the laboratory controls (LC). Amount of food and water supplementation and environmental conditions were simulated as in the flight group. After 3 months, thyroids and testis, including epididymis, were sampled from 1 WT and 2 TG mice housed in MDS (GC) as was stated above. Further, samples were also obtained from both LC and GC groups (n = 3 each).

### A. Thyroids

#### Reagents

Anti TSHR, anti-caveolin 1, horseradish peroxidase (HRP)-conjugated secondary antibody, fluorescein isothiocyanate (FITC)-conjugated secondary antibody were obtained from Santa Cruz Biotechnology, Inc. (CA, USA). SDS-PAGE molecular weight standards were purchased from Bio-Rad Laboratories (Hercules, CA, USA). Chemiluminescence kits, TSH and cAMP EIA kits were purchased from Amersham (Rainham, Essex, UK), Sigma-Aldrich Corporation (St. Louis, MO, USA) and CABRU SAS (Milan, Italy), respectively.

#### Thyroids analyses

After isolation, the left lobes were immediately frozen for subsequent morphological analysis while the right lobes were divided into 3 fragments: two fragments were treated with 10^−7^ or 10^−8^ M TSH for 1 hour, while the other fragment was untreated and used as control. After TSH stimulation, the fragments were fixed with absolute ethanol for 10 min at room temperature and centrifuged for 20 min at 3000×g. The supernatants were used for cAMP levels determination with an EIA kit according to manufacturing instructions. The pellets were used to quantify protein amount and for immunoblotting analysis.

#### Thyroid tissues

The thyroid lobes were fixed in 4% neutral phosphate-buffered formaldehyde solution for 24 h. The thyroids were dropped with essentially random orientation in paraffin. The paraffin blocks were sectioned into 4-µm-thick sections. All sections were mounted on silan-coated glass slides. Each slide contained a pair of sections at a distance equal to 140 µm. Sections between pairs 7 and 14 were sampled having excluded the first and the last. Then, sections 7, 9, 11 and 13 were used for morphological analysis whereas sections 8, 10, 12 and 14 were used for immunofluorescence analysis.

Tissue sections were deparaffinized and rehydrated through a series of xylene and ethanol washes.

#### Morphological analysis

The sections were stained by the hematoxylin-eosin (Chroma-Gesellschaft, Germany) staining method and investigated by using an inverted microscope, EUROMEX FE 2935 (ED Amhem, The Netherland), equipped with a CMEX 5000 camera system (4× and 40× magnification). The analysis of the tissue section size was performed by ImageFocus software.

#### Immunofluorescence analysis

After 3 washes with phosphate-buffered saline (PBS), sections were incubated with 2 µg/ml anti TSHR or caveolin1 primary antibodies diluted in a 0.5% solution of bovine serum albumin in PBS overnight at 4°C. The slides were washed 3 times with PBS and incubated with fluorochrome-conjugated secondary antibodies for 1 hour at room temperature. After 3 washes with PBS, the slides were mounted with glycerol and coverslips.

#### Pellet samples

The pellets were homogenized with a dounce homogenizer maintaining the temperature at +4°C throughout all the procedure. The suspension was used in part for protein dosage and in part for immunoblotting analysis.

Proteins analysis. The proteins were quantified according to Lowry et al. [49].

#### Western immunoblotting

About 30 µg of pellet proteins were submitted to SDS-PAGE electrophoresis in 10% polyacrylamide slab gel for TSHR and 12% slab gel for caveolin-1 detection. Electrophoresis image analysis was performed on gels stained with Coomassie-blue. Proteins were transferred into nitrocellulose for 90 min as previously described [50]. The membranes were blocked for 30 min with 0.5% no-fat dry milk in PBS (pH 7.5) and incubated overnight at 4°C with the specific antibody. The blots were treated with HRP-conjugated secondary antibodies for 90 min. Visualization was performed with the enhanced chemiluminescence kit. The apparent molecular weight of the proteins was calculated according to the migration of molecular size standards. The area density of the bands was evaluated by densitometry scanning and analyzed with Scion Image.

### B. Testis

Testes, including epididymis, were removed bilaterally. The right samples were quickly frozen in liquid nitrogen and stored using dry ice. They were then shipped to Osaka University, Japan. The left testes were divided in two halves: one was fixed in 4% paraformaldehyde and stored in 80% ethanol, the other was quickly frozen in liquid nitrogen and stored in dry ice. Specimens were then shipped to the Dip.Te.Ris, University of Genoa, Italy.

The fixed samples were processed for embedding in paraffin wax. Serial sections were cut (6 µm thick) and mounted on chrome alum gelatin-coated slides. They were then dewaxed, rehydrated and submitted to histological (Ematoxylin-Eosin staining) and immunohistochemical procedures following the indirect immunofluorescence technique [51]. After exposure in a moist chamber to normal rabbit or goat serum (diluted 1∶50; Santacruz Biotechnology Inc.) at 20°C, the unwashed sections were incubated overnight at 4°C with the following antisera: antibody to 3βHSD, raised in goat, 17βHSD, raised in rabbit, and HSP70 raised in goat. All the antisera were used at a dilution of 1∶500 (Santa Cruz Biotechnology Inc.). After washing in PBS, a second layer of fluoresceine-isothiocyanate conjugated gamma-globulins, donkey anti-goat (dil. 1∶20, Santa Cruz Biotechnology Inc.) or goat anti-rabbit (following the source of the primary antisera was applied for 30 min in a moist chamber, at 20°C. Sections were rinsed in PBS, mounted with glycerol-PBS (1∶9) and examined under a Leica epifluorescence microscope. The specificity of the immunostaining was verified by omitting one of the steps of the immunohistochemical procedure, or by replacing the primary antiserum with non-immune rabbit serum or PBS. After removing the coverslip, the sections were incubated at 20°C with the PAP complex diluted 1∶100 for 1 hr. As an electron donor, 0.025% 3.3′ diaminobenzidine tetrahydrochloride in PBS buffer containing 0.01% H_2_O_2_ was used.

#### RNA isolation and RT-Q-PCR

The frozen samples (in Genoa) were used for RNA extraction. Total RNA was isolated by the acid phenol-chloroform procedure using the Trizol reagent (Sigma) according to the manufacturers' instructions. Quality of isolated RNA was checked by electrophoresis on 1.5% agarose gel. Concentrations and purities of the isolated RNA were assessed by absorption spectroscopy. Only high purity samples (OD 260/280N1.8) were further processed. Aliquots of 1.5 µg RNA were reverse-transcribed into cDNA using 200 units RevertAid H Minus M-MuLV Reverse Transcriptase (Fermentas Italy, M-Medical, Milan), in presence of 200 pmol of poly-T18mer (TIB Mol Biol, Italia), 1 mM dNTPs (Fermentas) at 42°C for 60 min in a reaction volume of 20 µl. The cDNA was used to amplify the genes of interest using a Chromo 4™ System real-time PCR apparatus (Biorad Italy, Segrate, Milan). Proper aliquots of the RT mixture were diluted to a final volume of 20 µl in presence of iTaq SYBR Green Supermix with Rox (Biorad) and 0.25 µM of each specific primer pairs (TibMolBiol, Genoa, Italy). The primer pairs used and their references are shown in Table 2. Thermal protocol consisted of 3-min initial denaturation at 95°C followed by 40 cycles: 5 s at 95°C and 20 s at 60°C. A melting curve of PCR products (55–94°C) was also performed to rule out the presence of artifacts. Relative quantification of each gene expression was calculated according to comparative Ct method [52] using the Biorad software tool Genex-Gene Expression MacroTM. Expression of the genes of interest was normalized using the expression levels of GAPDH as housekeeping gene and the normalized expression was then expressed as relative quantity of mRNA (relative expression) with respect to laboratory and ground control samples.

**Table 2:** Primer Sequences.

**AR**	FW	5′- gCA gCT TgT gCATgTggT CA
	Rev	5′ – AATACCATCAgTCCCATCCAggAA
**LHR**	FW	5′- CCA TgggACgCTAATCT
	Rev	5′- gCAATTTggTggAAgAgACA
**FSHR**	FW	5′- CCTCTgCCAAgATAgCAAggTg
	Rev	5′ – CTCCAggTCCCCAAATCCAgA
**IL-1β**	FW	5′- CAggCAggCAgTATCACTCA
	Rev	5′- ggTgCTCATgTCCTCATCCT

Primer's sequence for androgen receptor is from Yang et al. [29]. Primer's sequences for lutenizing hormone receptor (LHR), follicle stimulating hormone receptor (FSHR), and interleukin-1beta (IL-1β) were designed by C. Barmo.

#### Sperm counts

After the mice were killed, the right epididymes were stored at −80°C in the Applied Physiology Laboratory at Osaka University until analyses. The frozen epididymes were submerged in 3-ml of PBS containing 1% protease inhibitor (Upstate) and incubated at 37°C for 5 min. The epididymes were minced with scissors and pressed using forceps moderately, to release all of the sperm into the solution. Sperm counting was performed using a Burker-Turk hematocytometer. Briefly, the sperm-containing solution was diluted 1∶2 with PBS. Then, 250 µl of sample-containing solution was placed on the hematocytometer and the sperms in two of the four arbitrarily chosen sections were counted under a microscope (Olympus, Tokyo). The counting was repeated three times for each sample. Data for sperm counts were expressed as the number of sperm per epididymis.
